# The effect of the COVID-19 pandemic on accrual-based earnings management: Evidence from four most affected European countries

**DOI:** 10.1016/j.heliyon.2024.e29890

**Published:** 2024-04-18

**Authors:** Alpaslan Yaşar, Neriman Yalçın

**Affiliations:** Department of Business, Adana Alparslan Türkeş Science and Technology University, 01250, Adana, Turkiye

**Keywords:** COVID-19 pandemic, Accrual-based earnings management, Income increasing/decreasing earnings management

## Abstract

This study aims to investigate the effect of the COVID-19 pandemic on accrual-based earnings management (EM) based upon a sampling of 938 listed firms from four selected European countries (United Kingdom, Italy, Spain, and Türkiye) during 2016–2020. We find that firms engage more in accrual-based EM through income-increasing and income-decreasing accruals in the year of the COVID-19 pandemic relative to the period before the pandemic. These findings imply that financial reporting reliability and usefulness decreased in the period of the pandemic. Our results are robust to some sensitivity checks.

## 1 Introduction

In December 2019, coronavirus disease 2019 (COVID-19) emerged in Wuhan, China, and has spread to a large number of countries around the world [1,2]. On January 30, 2020, the World Health Organization (WHO) announced a state of emergency due to the coronavirus disease and, after a short time, characterized it as a pandemic on March 11, 2020 [3].

COVID-19 lockdowns have had negative impacts on the national and global economies and financial system due to uncertainty, demand and supply shocks, and rational assessment of investors that firms’ profits would decline [4]. Market valuations of firms are adversely affected due to demand and supply shocks [5]. For many companies worldwide, the adverse impacts of COVID 19 have led to issues with cash and a deterioration of the balance sheet [6]. Like all great economic and financial crises, firms have run into a liquidity crisis and equity erosion caused by the COVID-19 shock [7]. Because of these negative effects, firms may engage in more accrual-based income-increasing earnings management (EM) practices based on opportunistic incentives to avoid declines in earnings [8].

EM is the manipulation of financial reports to mislead some stakeholders about the true financial performance of a company [9,10]. Managers pressured by the bad market conditions during COVID-19 may have engaged in EM using discretionary accruals to achieve their objectives [11]. Managers of companies with high financial distress levels may engage in income-increasing accrual-based EM to present the company in the most favourable light [12]. Additionally, the negative effects of COVID-19 may lead companies to manage their earnings upwards to show that they are not in a worse position than their competitors [6,13]. On the other hand, managers may be involved in a big bath by income-decreasing discretionary accruals in the current period to increase earnings in future years [8,13]. Additionally, managers of distressed companies may engage in income-decreasing EM practices and adopt a more conservative accounting approach during the distress period, either to reduce litigation risk voluntarily or because of pressure from auditors or lenders [14]. In this respect, it is essential to investigate whether these negative COVID-19-related factors provide substantial opportunities for companies to engage in EM. Conversely, if managers increase the transparency of their financial reporting to improve the trust of investors, companies engage in less EM during COVID-19 [15]. Therefore, under the assumption that investors use the information in financial reports for their decision making, it is essential to investigate EM practices in the COVID-19 period.

The findings of existing studies on the relationship between the COVID-19 pandemic and EM are insufficient and conflicting. Despite the increasing research in recent years on whether companies engage in EM during the pandemic, there have been few studies investigating the effect of COVID-19 on both aspects of accrual-based EM. Few studies (Lassoud and Knachel, 2021) [13] have examined separately the impact of COVID-19 on accrual-based EM through increasing (positive) and decreasing (negative) income in European samples. In addition, our study differs from previous studies in using both one-stage and two-stage regression models in EM estimations. Also, while previous studies [8,11,[16], [17], [18], [19], [20], [21]] concentrate on a single-country level, this study presents the analysis at the multi-country level. Moreover, our study differs from prior research regarding the selected countries. Due to the research gaps, it is important to investigate the direction of EM (positive or negative discretionary accruals) in the COVID-19 era. Thus, the research questions of our study are the following: 1) Do companies manage their earnings during the pandemic year? 2) If so, what is the direction of EM during the pandemic year?

This study aims to investigate the effect of the COVID-19 pandemic on accrual-based EM. Unlike most previous studies, we investigate whether companies engage in income-increasing or income-decreasing EM during the COVID-19 pandemic. Thus, we examine a sample of 938 firms listed in selected most affected European countries, such as the United Kingdom, Italy, Spain, and Türkiye, during the period 2016–2020. As of January 5, 2021, these are within the top ten countries that are most affected from COVID-19 in Europe [22] (https://www.who.int/publications/m/item/weekly-epidemiological-update---5-january-2021). Specifically, these countries are selected because of their high cumulative cases. As of June 1, 2020, these countries are listed among Europe's most affected five countries from the COVID-19 pandemic with confirmed cases [23] (https://iris.who.int/handle/10665/332281).

The paper makes two essential contributes. *First*, the present study adds additional evidence to the existing literature that very few studies (Lassoud and Knachel, 2021) [13] have investigated the impact of COVID-19 on both sides of accrual-based EM (i.e., positive and negative) at a multi-country level. Thus, our study helps to understand whether firms engage in EM through opportunistic incentives to avoid decline in earnings or take a big bath during the pandemic. *Second*, the results contribute to understanding the reliability and usefulness of accounting information during the pandemic.

The remaining of the research is constructed as the following structure. Section 2 provides the literature and the hypotheses. The methodological setting is explained in Section 3. The experimental findings of the study are documented in Section 4. The robustness tests are given in Section 5 and the conclusion discussed in the last section of the study.

## 2 Literature review and hypothesis development

### 2.1 Earnings management during crises

The financial crisis of 2008 and the outbreak of COVID-19 in 2020 are two major events that negatively affected global financial markets [24]. In contrast to the financial crisis of 2008, the COVID-19 crisis is truly exogenous (not the result of the unraveling of previous financial imbalances), is truly uncertain (depends on unpredictable non-economic factors), and is truly global (many countries, particularly in Asia, did not actually experience the 2007–2009 crisis) [25]. Cortes et al. document that the spillover effects of the subprime and COVID-19 crises have been markedly dissimilar in capital markets [26]. Despite their different origins and dynamics, the financial market effects of the two crises’ are comparable due to similar financial contagion and spillovers [27]. The COVID-19 pandemic has a considerably substantial economic impact than that of the global financial crisis [28]. Considering that the pandemic had more substantial implications than the 1997 Asian and the 2008 global crises, studies on the pandemic issues may reveal further implications for science [8]. Therefore, prior research investigating the effect of COVID-19 on EM has used economic crises as a background for their studies.

Companies may have an incentive to engage in EM in order to report high financial performance during the crisis and to overcome the slowdown, or they may adopt a less EM strategy, giving the market clear signals about the actual performance of the economy [15]. Given investors' tendency to attribute discretionary accruals as managerial opportunism instead of effective signaling, their confidence in financial reporting quality naturally declines in a crisis [29]. The evidence about the impact of discretionary accruals in times of economic crises regarding “opportunism versus informativeness” [30] is scarce. Assuming that financial crises exacerbate EM, there is evidence of both income-increasing and income-decreasing EM [31]. Some research [32,33] document that firms engage in income-increasing EM during a crisis, whereas others [30,[34], [35], [36], [37], [38]] find that firms apply income-decreasing EM to take a big bath. For example, Chia et al. [35] revealed that service-based firms engaged in income-decreasing EM for big-bath accounting in the Asian crisis. Habib et al. [30] investigated financially distressed firms engaged in EM during the 2008 crisis in the New Zealand sample. They found that financially distressed firms apply income-decreasing EM for reasons including poor investor confidence. On the contrary, some research has documented opposite findings [15,39,40] or mixed evidence [41,42] of the association between EM and crises. During crisis years, firms may engage less in EM activities due to increased scrutiny by auditors, creditors, and other stakeholders [35], higher litigation risk and higher demand for conservative earnings [40], and managers’ adoption of higher transparency in financial reporting to restoring the trust of investors and increasing liquidity [15]. Overall, there is no conclusive evidence from prior studies on financial crises and EM.

### 2.2 Earnings management during the COVID-19 pandemic

Managers under high pressure due to adverse market conditions during the pandemic may have used discretionary accruals to manage earnings to meet their targets [11]. Managers may use discretionary accruals for opportunistic EM, or they may use discretionary accruals as a tool of signaling a company's prospects to the market [29]. The behaviour of managers acting in line with their personal interests is the subject of agency theory [43,44]. According to this theory, managers manage EM during a pandemic or financial crisis to strategically manipulate this force majeure to serve their interests [13]. The opportunistic perspective (agency theory), which misleads external stakeholders about the underlying economic performance of the firm, has been the focus of most previous research on EM [45]. Some studies [8,13,[16], [17], [18],^46^,^47^] provide empirical findings on the relationship between the pandemic and EM consistent with opportunistic EM. In this context, Xiao and Xi [16] reveal that companies in the areas hardest hit by COVID-19 engage in accruals management rather than real EM. Yan et al. [18] investigate the relationship between the pandemic and EM using a sample of Chinese firms. They reveal that COVID-19 increased the accrual and real-based EM practices. Taylor et al. [47] investigate how the quality of financial reporting is affected during the pandemic on a sample of listed European banks. Their results indicate that EM levels increased during the pandemic years. Hsu and Yang [17] investigate the mitigating impact of corporate governance on financial reporting quality in firms listed in UK during the pandemic. Their results document a decline in the quality of the financial reporting and specifically, they find evidence of more EM through real activities during the COVID-19. Chen et al. [48] find an increase in accrued and real EM of Taiwan-listed firms in the period of COVID-19. Alternatively, EM can be informative or “signalling” rather than opportunistic in nature [41]. Firms with increased liquidity needs during a crisis tend to improve the financial reporting quality as a signal to the financial statement users and to obtain external financing [39]. In addition, according to stakeholder theory, the success of a company depends on its management to satisfy the interests of its key stakeholder groups by managing the relationship between them well [49]. Contradicting the agency theory, which arguing that a contractual relationship exists between managers and shareholders, the stakeholder theory, in essence, emphasizes that managers should be accountable to shareholders [50]. This theory states that managers should make decisions considering all stakeholders' interests in a firm [51]. One of the main incentives of EM during a pandemic is to rebuild stakeholder trust [52]. Therefore, managers avoid using a strategy such as EM to protect company's reputation and prevent conflicts with stakeholders [53]. Some studies have found that EM decreases during COVID-19, in line with signaling or stakeholder theory. Ali et al. [46] document that firms were less involved in EM in the period of COVID-19, consistent with findings of previous research [15,39,40] which conclude that EM decreases in times of the economic or financial crisis.

Firms may manage earnings through income-increasing or income-decreasing EM practices based on an opportunistic perspective for various reasons (such as avoiding earnings declines and taking a big bath) in the pandemic [8,29]. Managers may engage in EM practices that increase income in the pandemic period relative to the pre-pandemic period to avoid the possibility of firms experiencing declines in earnings or incurring losses in the pandemic year [8]. Similar to previous studies on crises, research shows that firms engage more in EM through income-increasing during the pandemic period [13,33]. Khanchel and Lassoued [19] document that socially responsible firms engage in income-increasing EM. Also, Yan et al. [18] and Aljughaiman et al. [11] reveal that managers used income-increasing accrual-based EM during the COVID-19.

Based on the arguments described above, the first hypothesis of this study is as follows:

H1 Companies engage more in EM through income-increasing accruals during the pandemic era of COVID-19.

On the other hand, investors’ bias to link weak financial performance with bad luck in periods of high uncertainty encourages managers to take a big bath by opportunistically reporting more negative discretionary accruals during such periods [31]. Management may try to take advantage of the pandemic year to have a big bath by engaging in more income-decreasing EM. However, if managers believe that the economic effect of the pandemic will be long-term, then managers may not engage in big bath accounting during the pandemic period [8]. In this context, Liu and Sun [8] examine the impact of the pandemic on EM in the USA. Similar to empirical evidence from prior research on financial crises [35,36], their findings indicate that companies engage in EM that decrease income with the incentives of big-bath during the pandemic. Garfatta et al. [54] show that Tunisian firms engage in income-decreasing EM during the pandemic. Similarly, Lizińska & Czapiewski [21] document that Polish companies deal with the ‘big bath’ EM in the pandemic. Thus, our second hypothesis is as follows:

H2 Companies engage more in EM through income-decreasing accruals during the pandemic era of COVID-19.

## 3 Research design

### 3.1 Sample selection

This study is based on data from annual reports of publicly traded companies in four selected countries (Italy, Spain, Turkey, and the United Kingdom) for the period from 2016 to 2020. We consider 2020 as the pandemic year and 2016–2019 as the pre-pandemic period. We gathered the data on firm-level variables from Eikon's database. The initial sample comprises 15,820 firm-year observations of 3164 listed firms for 2016–2020. As in previous studies [8,16,46], the initial sample is reduced to 9030 firm-year observations by excluding financial firms. Next, we exclude missing data. Thus, the final sample consists of 4690 firm-year data of 938 quoted firms for the fiscal years 2016–2020. The distribution of the sample by country is reported in Table 1.

**Table 1:** Sample distribution by country.

	Italy	Spain	Türkiye	United Kingdom
Publicly listed companies (2016–2020)	426	254	385	2099
Financial companies	(105)	(101)	(116)	(1036)
Companies with incomplete data	(122)	(45)	(58)	(643)
The final number of firms (n = 938)	**199**	**108**	**211**	**420**

### 3.2 Variables

Table 2 shows the detailed descriptions of the dependent, independent, and control variables. Discretionary accruals are operationalized as a proxy for EM. We calculated discretionary accruals (*DA*_*it*_) by subtracting non-discretionary accruals (estimated using the model of Kothari et al. [55]) from total accruals (using the cash flow statement method). Specifically, we estimate discretionary accruals from the Kothari et al. [55] model by industry and year for all firms. Kothari et al. [55] proposed two modifications of the Jones models: an intercept to mitigate heteroskedasticity, and the lagged return on assets (ROA) variable. We estimate the model in equation (1) by using the lagged ROA variable to reduce heteroscedasticity. We measure the level of accrual-based accruals by the absolute amount of discretionary accruals (|*DAit*|) [15,[56], [57], [58]]. We also separate the normal (signed) value of estimated discretionary accruals in positive (${DA}_{it}^{+}$), and negative (${DA}_{it}^{-}$) discretionary accruals, to determine whether companies manage discretionary accruals to inflate or decrease income during the COVID-19 pandemic year.^1^

(1) $${TA}_{it}/A_{it-1}=\alpha_{1}\left(1/A_{it-1}\right)+\alpha_{2}\left({\Delta REV}_{it}-{\Delta REC}_{it}\right)/A_{it-1}+\alpha_{3}\left({PPE}_{it}\right)/A_{it-1}+{\alpha_{4}ROA}_{it}/A_{it-1}+\varepsilon_{it}$$

where:

**Table 2:** Definition of the variables.

Variable	Definition
*Dependent variable*	
*|DA|*	is the absolute value of discretionary accruals measured by the model of Kothari et al. (2005)
*DA*^*+*^	is the positive discretionary accruals
*DA*^*-*^	is the negative discretionary accruals
*Independent variables*	
*Covid19*	is equal to 1 if the observation is from the pandemic year 2020 and 0 otherwise
*Control variables*	
*SIZE*	is the natural log (LN) of total assets
*LEV*	is the total liabilities divided by total assets
*OCF*	is the operating cash flows deflated by t-1 total assets
*ROA*	is the return on assets deflated by t-1 total assets
*BIG4*	equals 1 if the audit firm is Deloitte, PwC, E&Y, or KPMG, and 0 otherwise
*GROWTH*	is the growth in net sales

*TA* = total accruals for year t.

*A*_*it-1*_ = total assets for year t-1.

*ΔREV* = change in net revenues for year t.

*ΔREC* = change in net receivables for year t.

*PPE* = gross property, plant, and equipment for year t.

*ROA* = return on assets for year t-1

*ε* = residual for year t.

### 3.3 Research model

To determine whether the impact of the pandemic on accrual-based EM varies with the absolute (*|* ${DA}_{it}$ *|*), positive (${DA}_{it}^{+}$), and negative (${DA}_{it}^{-}$) value of discretionary accruals, we estimate the model in Eq. (2) using ordinary least squares (OLS) regression analysis:

(2) $${\;|DA}_{it}|=\beta_{0}+\beta_{1}{Covid19}_{it}+\beta_{2}{SIZE}_{it}+\beta_{3}{LEV}_{it}+\beta_{4}{OCF}_{it}+\beta_{5}{ROA}_{it}{{+\beta }_{6}BIG4}_{it}+\beta_{7}{GROWTH}_{it}+{\;fixed\;effects}_{it}+\varepsilon_{it}$$

The variables in equation (2) are defined in Table 2. We winsorized the variables in equation (2) at 1 % and 99 % percentiles to reduce the impact of the outliers. The data meet the test assumptions (normality of errors, heteroscedasticity, and multicollinearity) for the OLS model.

## 4 Empirical results

### 4.1 Descriptive statistics

Descriptive statistics of the variables for the pooled sample and the pre-pandemic and pandemic periods are presented in Table 3. Panel A shows the descriptive statistics for the accrual-based EM proxies *|DA|, DA*^*+*^*,* and *DA*^*-*^ before and during the pandemic period. The mean *|DA|, DA*^*+*^*,* and *DA*^*-*^ (0.0327, 0.0312, and −0.0345, respectively) are greater in COVID-19 than pre-pandemic times (0.0104, 0.0092, and −0.0136, respectively). These statistics document that companies involved in more accrual-based EM during the pandemic period.

**Table 3:** Descriptive statistics.

Panel A: Dependent variables							
Variables		Pooled sample (2016–2020)		Pre-pandemic (2016–2019)		Pandemic (2020)	
	N	Mean	SD	Mean	SD	Mean	SD
*|DA|*	4690	0.0116	0.0418	0.0104	0.0402	0.0327	0.1748
*DA*^*+*^	3216	0.0111	0.0418	0.0092	0.0415	0.0312	0.1834
*DA*^*-*^	1474	−0.0128	0.0418	−0.0136	0.0359	−0.0345	0.1648
Panel B: Control variables							
*SIZE*	4690	19.8497	2.3402	19.7598	2.3001	20.2090	2.4627
*LEV*	4690	0.2941	0.2012	0.2903	0.2001	0.3082	0.2048
*OCF*	4690	0.0605	0.4377	0.0779	0.4073	−0.0094	0.5370
*ROA*	4690	0.7860	6.5280	0.9779	7.2523	0.0183	1.4034
*BIG4*	4690	0.6632	0.4727	0.6707	0.4700	0.6333	0.4822
*GROWTH*	4690	0.2242	2.5025	0.2561	2.7676	0.0964	0.8099

**Notes:** This table reports the mean and standard deviation of the variables for the pooled sample and pre-pandemic and pandemic periods. The variables are defined in Table 2, and are winsorized at 1 percent and 99 percent to reduce the influence of the outliers.

### 4.2 Univariate analysis

Table 4 reports the parametric *t*-test results of our dependent variables (*|DA|*, *DA*^*+*^*,* and *DA*^*-*^). The univariate test findings indicate that the difference in the mean between the pre-pandemic and pandemic eras is statistically significant for *|DA|*, *DA*^*+*^*,* and *DA*^*-*^. The results show that accrual-based EM increases during the COVID-19 era, and companies engage in income-increasing and income-decreasing EM.

**Table 4:** Univariate test results.

	Pre-pandemic (2016–2019)	Pandemic (2020)	Mean Difference	t-value	p-value
*|DA|*	0.0104	0.0327	−0.0223	−7.114***	0.000
*DA*^*+*^	0.0092	0.0312	−0,0220	−5.545***	0.000
*DA*^*-*^	−0.0136	−0.0344	0.0208	3.812***	0.000

**Notes:** Significance levels are two-tailed.

*, **, *** Significant at 0.10, 0.05, and 0.01, respectively.

### 4.3 Correlation analysis

The Pearson matrix is presented in Table 5. The coefficients of correlation are low and for the most part below 0.15. This indicates that multicollinearity between the variables does not exist.

**Table 5:** Pearson correlation matrix.

Variables	*|DA|*	*Covid19*	*SIZE*	*LEV*	*OCF*	*ROA*	*BIG4*	*GROWTH*
*|DA|*	1							
*Covid19*	0.103**	1						
*SIZE*	−0.047**	0.077**	1					
*LEV*	−0.061**	−0.491**	0.078**	1				
*OCF*	−0.040**	−0.080**	0.096**	0.016	1			
*ROA*	0.098**	−0.059**	0.085**	−0.012	0.116**	1		
*BIG4*	−0.047**	−0.032*	0.460**	0.013	0.108**	0.042**	1	
*GROWTH*	0.014	−0.026	−0.045**	0.019	−0.025	−0.001	−0.051**	1

### 4.4 Multiple regression analysis

The multivariate regression results of the COVID-19 pandemic and accrual-based EM are presented in Table 6.

**Table 6:** The regression results of COVID-19 and accrual-based EM.

Variables	*|DA|* (1)	*DA*^*+*^ (2)	*DA*^*-*^(3)
*Covid19*	0.025^∗∗∗^	0.032^∗∗∗^	−0.025^∗∗∗^
	(5.715)	(6.283)	(-3.600)
*SIZE*	−0.002^∗∗∗^	−0.001	0.004^∗∗∗^
	(-2.450)	(-1.576)	(2.790)
*LEV*	−0.001	0.004	0.010
	(-0.104)	(0.450)	(0.749)
*OCF*	−0.013^∗∗∗^	0.029^∗∗∗^	0.168^∗∗∗^
	(-3.495)	(7.768)	(23.362)
*ROA*	0.002^∗∗∗^	0.003^∗∗∗^	0.004^∗∗∗^
	(6.148)	(12.440)	(8.337)
*BIG4*	−0.003	−0.002	0.007
	(-0.693)	(-0.393)	(0.215)
*GROWTH*	0.001	0.001	0.005^∗∗^
	(0.901)	(1.368)	(1.977)
Constant	0.048^∗∗∗^	0.030^∗^	−0.080^∗∗∗^
	(3.379)	(1.715)	(-3.517)
Industry FE	Yes	Yes	Yes
Year FE	Yes	Yes	Yes
Country FE	Yes	Yes	Yes
N	4690	3244	1446
Adj. *R*^*2*^	0.024	0.089	0.394
*F*	14.268	38.248	97.790

**Notes:** Significance levels are two-tailed.

*, **, ***Significant at .10, .05, and .01, respectively.

To test our hypotheses (H1 and H2), we take *|DA|*, *DA*^*+*^*,* and *DA*^*-*^ to be the dependent variables. When the (*|DA|*) is the dependent variable in equation (2), after controlling the factors that potentially influence discretionary accruals, we find that the (*|DA|*) is significantly and positively related with *Covid19* at the 1 % level. The findings indicate that firms engage more in accrual-based EM (income-increasing or income-decreasing EM) during the pandemic. This is consistent with the evidence in previous studies [13,16,18]. In addition, the results are similar to the prior findings of Persakis and Iatridis [32], Lisboa [61], and Tombetta and Imperatore [33], which document that financial crises have a significant effect on EM activities.

To test our hypotheses (H1 and H2), we use the (*DA*^*+*^) and (*DA*^*-*^) as independent variables in equation (2). The regression coefficient of *Covid19* in Column (2) of Table 6 is significantly positive (p < 0.01), suggesting that companies engage in income-increasing EM during the COVID-19 pandemic. The results are consistent with the Hypothesis H1. These findings are consistent with those of Lassoued and Khanchel [13] and Yan et al. [18]. Additionally, the results are in line with the findings of Trombetta and Imperatore [33], which indicate that managers involve in income-increasing EM activities when the financial crisis is of high severity. The results show that company managers may have involved in income-increasing EM practices through discretionary accruals for various purposes during the pandemic, as in financial crisis periods. Therefore, it is possible that companies hit by the COVID-19 outbreak have managed earnings upwards to regain confidence of investors and to show that they are not in a worse position than its competitors [6,13]. On the other hand, Column (3) reveals that when income-decreasing discretionary accruals (*DA*^*-*^) are used as the independent variable, the coefficient of *Covid19* is significantly negative (p < 0.01), which indicates that firms engage in income-decreasing EM in the period of the pandemic. The results are also consistent with the Hypothesis H2 and those reported in Table 4. These findings are consistent with those of Liu and Sun [8], which shows that firms used income-decreasing EM for a big-bath approach during the pandemic. In addition, Chia et al. [35] and Rusmin et al. [36] found similar results for the Asian financial crisis. These results suggest that managers may opportunistically engage in more negative discretionary accrual (big-bath) reporting during periods of high uncertainty, such as pandemics. Thus, managers can take a big bath in the current period by managing earnings downwards through income-reducing discretionary accruals to increase earnings in future years. Overall, these results show that companies involve in accrual-based 10.13039/100006138EM through income-increasing (positive) and income-decreasing (negative) accruals in the COVID-19 pandemic year than before, supporting the opportunistic perspective.

### 4.5 Robustness checks

We performed several robustness tests. *First*, researchers may be unsure of the result if they estimate discretionary accruals for a skewed sample by using the performance-matched model of Kothari et al. [55]. Therefore, we also employ the adjusted Jones model [62] to capture discretionary accruals and repeat the regression analysis on the three models of discretionary accruals (*|DA|*, *DA*^*+*^, and *DA*^*-*^) in Eq. (2). The *Covid19* coefficient in all columns of Table 7 shows evidence that *Covid19* is associated with *|DA|*, *DA*^*+*^, and *DA*^*-*^, which supports the findings in Table 6.

**Table 7:** Robustness checks performed with the model of modified Jones.

Variables	*|DA|* (1)	*DA*^*+*^ (2)	*DA*^*-*^(3)
*Covid19*	0.010***	0.009***	−0.013***
	(5.397)	(3.189)	(-6.025)
*SIZE*	−0.001***	−5.920	0.003***
	(-4.292)	(-0.128)	(7.784)
*LEV*	0.002	0.002	−0.003
	(0.482)	(0.375)	(-0.701)
*OCF*	0.023***	0.036***	−0.003*
	(15.601)	(17.574)	(-1.753)
*ROA*	7.379	4.115	4.323
	(0.741)	(0.353)	(0.230)
*BIG4*	−0.006***	−0.006***	0.007***
	(-4.112)	(-2.967)	(3.965)
*GROWTH*	0.001	0.001	0.001
	(1.303)	(1.550)	(0.504)
Constant	0.036***	0.008	−0.069***
	(6.088)	(0.910)	(-9.627)
Industry FE	Yes	Yes	Yes
Year FE	Yes	Yes	Yes
Country FE	Yes	Yes	Yes
N	4690	3244	1446
Adj. *R*^*2*^	0.076	0.111	0.085
*F*	44.509	46.762	16.200

**Notes:** Significance levels are two-tailed.

*, **, ***Significant at 0.10, 0.05, and 0.01, respectively.

*Second*, similar to some previous studies [13,18] on COVID-19, we exclude the sample in 2019 and conduct a robustness test because the annual reports of the listed companies for 2019 may be affected by the busy audit season from January to April 2020. The findings given in Table 8 are generally consistent with the findings in Table 6, indicating that companies engaged in more accrual-based EM during the COVID-19 pandemic year.

**Table 8:** Robustness test results after excluding the sample in 2019.

Variables	*|DA|* (1)	*DA*^*+*^ (2)	*DA*^*-*^(3)
*Covid19*	0.024***	0.033***	−0.023***
	(4.567)	(5.348)	(-2.860)
*SIZE*	−0.002***	−0.002*	0.004***
	(-2.359)	(-1.608)	(2.680)
*LEV*	−0.002	0.004	0.016
	(-2.359)	(0.361)	(0.318)
*OCF*	−0.013***	0.028***	0.173***
	(-3.124)	(6.608)	(21.879)
*ROA*	0.001***	0.004***	0.004***
	(4.281)	(10.761)	(7.603)
*BIG4*	−0.003	−0.002	0.009
	(-0.701)	(-0.344)	(1.261)
*GROWTH*	0.001	0.001	0.005*
	(0.775)	(1.199)	(1.881)
Constant	0.057***	0.038*	−0.090***
	(3.222)	(1.706)	(-3.438)
Industry FE	Yes	Yes	Yes
Year FE	Yes	Yes	Yes
Country FE	Yes	Yes	Yes
N	3752	2546	1206
Adj. *R*^*2*^	0.019	0.086	0.404
*F*	9.363	28.946	85.228

**Notes:** Significance levels are two-tailed.

*, **, *** Significant at 0.10, 0.05, and 0.01, respectively.

*Third*, the two-stage regression procedure of existing accrual models can produce biased estimates and lead to Type I and II errors [63]. Chen et al. [64] proposed a single-stage regression model (the independent variables used in the first stage are included as additional independent variables in the second-stage regression model) as a solution to the potential biases introduced by the two-stage procedure. Therefore, we estimate the model in equation (2) using a single-step regression model, and the findings are tabulated in Table 9. As shown in Table 9, the results are still robust when we use single-stage regression.

**Table 9:** Robustness test results using a single-step regression model.

Variables	*Accrual-based EM (Total accruals)*
*Covid19*	−0.047**
	(-2.187)
*SIZE*	−0.011***
	(-2.505)
*LEV*	0.004
	(0.087)
*OCF*	0.184***
	(8.719)
*ROA*	0.004***
	(3.058)
*BIG4*	0.021
	(1.026)
*GROWTH*	0.003
	(0.800)
Constant	0.222***
	(2.610)
Industry FE	Yes
Year FE	Yes
Country FE	Yes
N	4690
Adj. *R*^*2*^	0.034
*F*	13.255

**Notes:** Significance levels are two-tailed.

*, **, ***Significant at 0.10, 0.05, and 0.01, respectively.

*Finally*, as in some studies [27,65,66] in the literature, we also take the natural log of one plus the number of accumulative confirmed cases^2^ of COVID-19 in the sample countries during 2020 to overcome clear bias through a dummy (that equals 1 for 2020 and 0 otherwise). We obtained qualitatively similar results consistent with the findings in Table 6 (These robustness results are not tabulated for reason of brevity).

## 5 Conclusions

The COVID-19 pandemic had negative effects on the performance of firms in almost all industries worldwide and led to volatility in global markets. The COVID-19 pandemic crisis may incentivise firms to implement income-increasing EM with opportunistic incentives to avoid a decline in profits or restore investor and stakeholder confidence, or implement income-decreasing big bath accounting. Therefore, we investigate whether firms engage in accrual-based EM during the pandemic year and, if so, what is the direction of EM during the pandemic year.

Based on sample of 938 listed companies of four selected European countries (United Kingdom, Italy, Spain, and Türkiye) for the 2016–2020 period, we find that firms engage more in both accrual-based income-increasing and income-decreasing EM. The results support the results of Lassoued and Khanchel [13] and Yan et al. [18], which indicate that firms engage in income-increasing EM during the COVID-19 pandemic. These findings are also consistent with the results of Liu and Sun [8], which find that firms used income-decreasing EM for a big-bath strategy during the pandemic year. Thus, our findings are robust to controls and indicate that companies engage in both income-increasing and income-decreasing accrual-based EM during the pandemic. Therefore, during periods of high uncertainty, such as pandemics, managers of firms apply EM more opportunistically than before the pandemic, leading to a decrease in financial reporting quality. The resulting decrease in the financial reporting quality impairs the relevance of accounting information for users in decision-making.

The study has several implications. *First*, our results are helpful for users of financial information to pay more attention to firms’ EM practices during a pandemic crisis. *Second*, our findings draw investors' attention to the fact that during crisis periods such as the pandemic outbreak, firms may resort to “big bath” practices, that is, to increase their losses by using discretionary accruals. *Third*, our results also highlight audit risks that may arise from companies' EM practices during the COVID-19 crisis; therefore, auditors should design audit procedures to reduce potential audit risks associated with the COVID-19 era.

This study has some limitations, similar to all studies. *First*, we select four developed and developing countries among the ten most affected countries by COVID-19. Therefore, including data from other developed and developing countries affected by COVID-19 would provide more insight into the results. *Second*, we consider the entire year of 2020 for the COVID-19 era. However, the results of this study may not entirely reveal the effect of the COVID-19, given that the first confirmed cases of COVID-19 in the sampled countries occurred on or after January 31, 2020. Third, we examine only the original reaction of firms during the COVID-19 period. However, the data from 2021 to 2022 can be used to determine the effect after COVID-19. *Fourth*, as in the previous literature, we adopt accrual-based EM and consider discretionary accruals as a proxy for EM. However, it should be noted that there are some criticisms that discretionary accruals are noisy proxies and are not the most appropriate indicator of EM. In addition, this study uses only accrual-based EM during COVID-19. Future studies may analyze this issue using different EM tools, such as real EM and classification shifting, which are more challenging to determine. Furthermore, quarterly data can be used to better understand the association between COVID-19 crisis and EM. Finally, as managerial psychological characteristics may influence EM [68,69], the impact of the pandemic on managerial psychology and its relationship to EM may be of interest to future research.

## Data availability statement

There is no research-related data stored in publicly available repositories, and the data will be made available upon reasonable request.

## Additional information

No additional information is available for this paper.

## CRediT authorship contribution statement

**Alpaslan Yaşar:** Writing – review & editing, Writing – original draft, Supervision, Methodology, Investigation, Formal analysis, Conceptualization. **Neriman Yalçın:** Writing – review & editing, Visualization, Investigation, Data curation, Conceptualization.

## Declaration of competing interest

The authors declare that they have no known competing financial interests or personal relationships that could have appeared to influence the work reported in this paper.

## Footnotes

1 However, firm-specific economic shocks may lead both to noise in discretionary accrual estimates and to a deterioration of the bias in discretionary accruals (for unsigned and signed accruals) [59,60].

2 Confirmed cases data are retrieved from https://www.worldometers.info/coronavirus/ [67].
